# How to get a nation walking: reach, retention, participant characteristics and program implications of Heart Foundation Walking, a nationwide Australian community-based walking program

**DOI:** 10.1186/s12966-017-0617-5

**Published:** 2017-11-21

**Authors:** Kylie Ball, Gavin Abbott, Michelle Wilson, Melanie Chisholm, Shannon Sahlqvist

**Affiliations:** 1 Deakin University, Geelong, Australia, Institute for Physical Activity and Nutrition (IPAN), School of Exercise and Nutrition Sciences, Melbourne Burwood Campus, 221 Burwood Hwy, Burwood, VIC 3125 Australia; 2 Heart Foundation, 155-159 Hutt St, Adelaide, SA 5000 Australia; 3 North Western Melbourne PHN, 369 Royal Parade, Parkville, VIC 3052 Australia

**Keywords:** Walking, Community, Program, Reach, Retention, Socioeconomic

## Abstract

**Background:**

Community-based walking programs represent a low-cost, accessible approach to increasing physical activity among inactive adults. However, recruiting participants from vulnerable and hard-to-reach groups remains a challenge. This study examined the reach, retention, sociodemographic and health characteristics, physical activity levels and motivators of participants in Heart Foundation Walking, a nationwide Australian community-based walking program.

**Methods:**

Descriptive cross-sectional analyses were undertaken with data from 22,416 participants aged 15+ years in the Heart Foundation Walking registration database in December 2015, and from four surveys of participants in 2010 (*n* = 2400), 2011 (*n* = 3274), 2012 (*n* = 4158) and 2015 (*n* = 1890).

**Results:**

Heart Foundation Walking reached participants in every geographic region of Australia, including remote and sparsely populated regions, and engaged sizeable proportions of the following at-risk participants: older than 60 years (>70%); with very low incomes (17–25%); who were overweight or obese (around 60%); and with one or more chronic disease or disease risk factors (57–81%). For all demographic groups, one-year retention rates were at least 75%. Seventy percent of participants met physical activity recommendations. Over 75% reported joining the program for health and fitness reasons while the most cited motivator for continuing was the social aspect (57–73%).

**Conclusions:**

Volunteer-run, group-based walking programs can have substantial reach and retention, in particular among those at risk for physical inactivity. The provision of opportunities for social interaction appears to be a key program element in promoting long-term participation, including among high-risk groups.

## Background

Given the benefits of physical activity to overall health and well-being, high population levels of inactivity remain of concern, in particular in vulnerable groups such as older adults or those experiencing socioeconomic disadvantage [1, 2]. Community-based programs represent a promising approach for engaging large numbers of people in the settings in which they live [3]. Walking is an activity involving low cost, risk of injury, and skill requirements. Group-based walking programs have been shown to be effective in increasing physical activity [4, 5] and improving health [6]. However, recruitment into such programs, particularly from high-risk groups, is challenging [7], and low retention is often problematic [8, 9]. Little is known about reach, retention rates, participant characteristics, or successful implementation elements of walking programs. Evaluations of ‘real-world’ large-scale physical activity programs in the community are also scarce, and have been highlighted as a priority [5]. Evidence of elements of successful walking group programs would inform future community-based physical activity programs with potential broad and equitable reach and impact.

Heart Foundation Walking (http://walking.heartfoundation.org.au/) is a voluntary group-based community walking program rolled out across Australia in 2007 to facilitate regular physical activity in safe, supportive social environments. There were 1287 registered groups, with varying numbers of participants, as of December 2015. The aim of this study was to examine the reach, retention, sociodemographic and health characteristics, physical activity levels and motivations for joining and remaining in the program.

## Methods

### Participants

Data were drawn from the Heart Foundation Walking national registration database as of 13th December 2015 (*n* = 22,416 participants aged ≥15 years; 20,753 walkers, 1663 Walk Organisers), and from four surveys of participants registered at the time of each survey. These were undertaken in 2010 (*n* = 1984 walkers and 416 Walk Organisers, representing response rates of 33 and 30%); 2011 (2796 walkers, 478 Walk Organisers, 44 and 45%); 2012 (3585 walkers, 573 Walk Organisers, 33 and 32%) and 2015 (1601 walkers, 289 Walk Organisers, 15 and 25%). Surveys were sent by email or mail in 2010, 2011 and 2012; and email only in 2015. The research was performed in accordance with the Declaration of Helsinki, and an exemption from Ethics review (ref 2015–245) was granted by the Deakin University Human Research Ethics Committee for analysis of this routinely collected de-identified data.

### Program

Heart Foundation Walking utilises local government and community health centre staff to support the program as part of their core business, with walks led by local volunteer Walk Organisers. It is promoted through low cost local media releases, posters and flyers in health and community centres, social media and limited digital advertising and bring a friend campaigns. Retention strategies, which are of no cost to participants and minimal cost to the organization, include a Walker Recognition Scheme which rewards participants upon reaching milestones; quarterly newsletters; group anniversary certificates; annual events such as large group walks and volunteer workshops; annual photo competitions and “golden shoe awards” for key supporters.

### Measures and analyses

Upon registration, all participants reported their demographic data and how they heard about the program. Based on residential address, participants were classified into one of 88 geographic regions (known as Statistical Areas Level 4: SA4) of Australia. Reach rates were calculated as the numbers of SA4s in which participants were located, and the number of participants as a proportion of the population in each SA4. Residential address was also used to determine Socioeconomic Index for Areas (SEIFA) [10], a marker of neighbourhood socioeconomic disadvantage.

In each survey, using standard items participants reported their age, household structure, gross household income category, height and weight (for calculating body mass index [BMI]), and if they had any specified chronic diseases or risk factors. The Active Australia survey, which assesses the total time in the last week spent walking (for recreation, exercise, or to get from place to place); in vigorous gardening/yard work (not used in calculating physical activity in these analyses); in other vigorous-intensity activities; and in other moderate-intensity activities, was used to assess physical activity. The Active Australia survey has acceptable reliability [11] and criterion validity [12]. Data were treated according to standard protocols [13], with a dichotomous variable created to indicate participation in at least 150 min/week of at least moderate intensity physical activity in at least five sessions. Using questions developed for the study, participants were asked to select from a list of responses, the main reasons they joined the program; and a separate open-ended question on the main reasons they were still participating. They were also asked to rate on a 6-point Likert scale how important they perceived three elements: the Walker recognition scheme; quarterly newsletters; and special events.

Because numbers of participants responding to more than one survey were relatively low, and not all surveys were ID-linked, survey data are presented as serial cross-sectional data. Descriptive analyses were used to examine reach and participant characteristics, retention, and reasons for participation. Where available, comparisons with the general population were made to examine representativeness of participants. Linear regression was used to examine differences in retention time by recruitment mode.

## Results

### Reach and participant characteristics

**Fig. 1:**
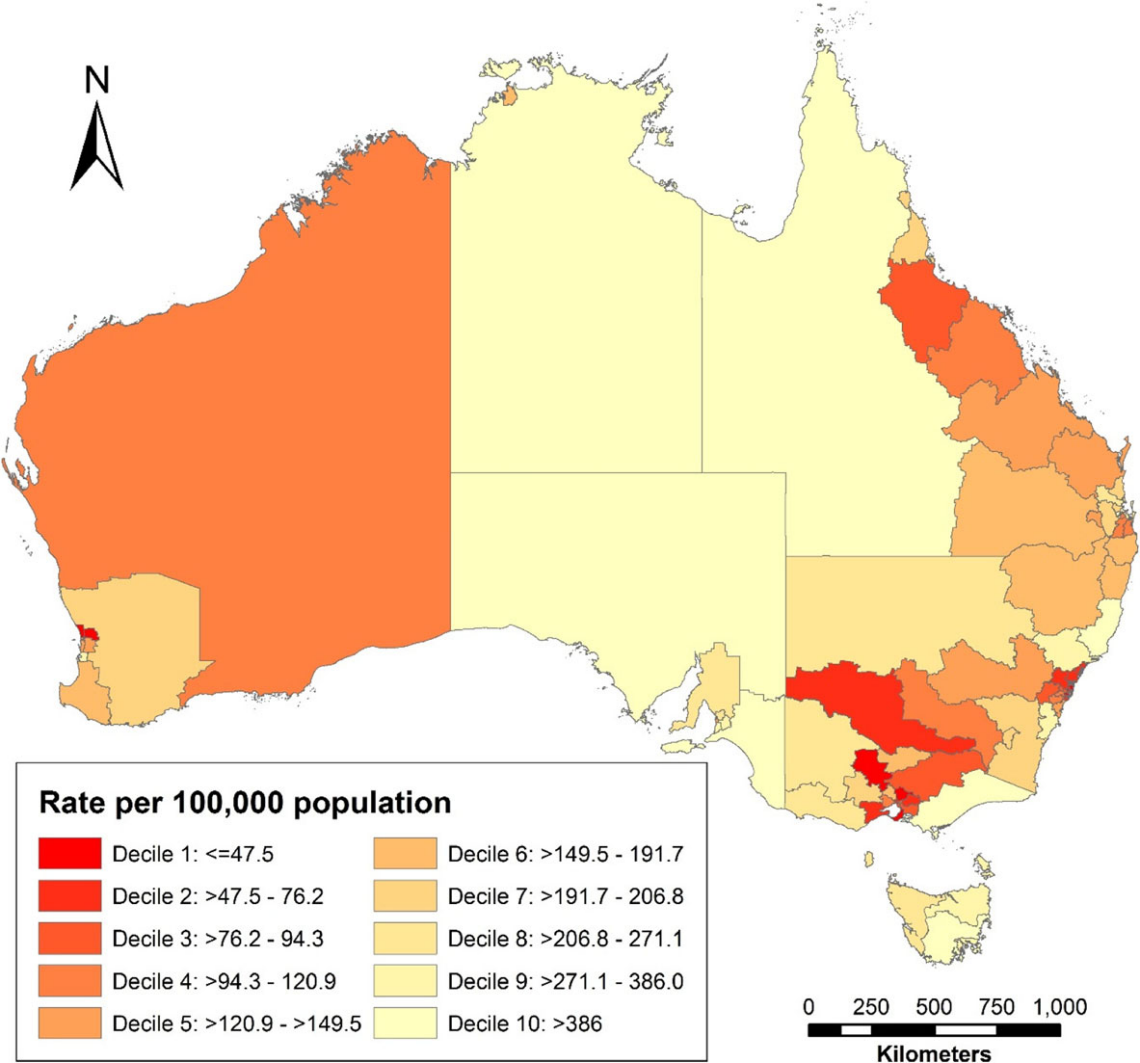
Participation rates per 100,000 population of Heart Foundation Walking across Australia (note Australia’s population reside predominantly in coastal cities, particularly in the east and south east, with sparser populations in other regions and inland)

Overall, walkers (*n* = 20,753) had participated in Heart Foundation Walking for an average 2.4 (SD = 2.7) years. Analysis of the national database indicated that Heart Foundation Walking participants were located in all 88 SA4 regions of Australia, with a median of 245 participants/region (range 8–804). The median number of participants as a proportion of the total population in each region was 148.4/100,000 residents (range 3.8–1000/100,000). Remote and sparsely populated regions were over-represented in those with high participation rates (Fig. 1).

For the years survey data were available, walkers had an average age of 64 years, and more than three-quarters were women (Table 1). Around a quarter lived alone, a figure substantially higher than that for the general population (9%) [14]. The income distribution of walkers was skewed towards low income categories. A sizeable proportion had an average annual household income of less than $25,000, a very low income threshold (for example, the mean household income in 2013–14 was $107,276) [15].

**Table 1:** Socioeconomic characteristics of walkers participating in each survey^a^

	2010 (*n* = 1984)	2011 (*n* = 2796)			2012 (*n* = 3585)			2015 (*n* = 1601)		
		All	Female	Male	All	Female	Male	All	Male	Female
Age (Mean, SD)	Data not available	63.9 (11.9)	62.9 (12.2)	67.5 (9.9)	64.3 (11.8)	63.5 (12.0)	67.3 (10.6)	64.6 (10.0)	63.6 (9.9)	68.4 (9.1)
Age group (%)	Data not available									
15–29		1.6	1.9	0.2	2.0	2.2	1.2	0.9	1.1	0.0
30–39		3.3	3.6	2.3	2.6	3.1	0.6	1.9	1.9	2.1
40–49		6.2	7.0	3.0	5.3	5.5	4.3	5.0	5.9	1.5
50–59		16.1	18.0	8.9	16.0	17.5	10.7	14.3	15.9	8.0
60–69		39.9	39.2	42.5	40.8	41.5	38.0	48.3	50.0	41.9
70–79		27.3	25.0	35.8	27.1	24.6	36.2	26.3	23.0	39.1
80+		5.6	5.1	7.3	6.3	5.6	9.0	3.3	2.2	7.3
Sex (%)	Data not available									
Female		79.2			78.5			79.5		
Male		20.8			21.5			20.5		
Household structure (%)										
Person living alone	25.7	27.6	30.4	17.1	26.7	28.7	19.3	25.7	27.4	19.3
Couple only	50.7	51.9	47.3	69.1	51.8	48.2	65.0	54.0	50.9	66.4
Couple living with their child(ren)	11.0	11.9	12.2	10.6	11.2	11.7	9.4	10.6	11.5	7.3
Single person living with their child(ren)	2.6	2.7	3.4	0.3	2.6	3.0	1.1	2.6	3.0	1.2
Adult living at home with parents	1.3	1.0	1.1	0.7	1.1	0.9	1.7	0.7	0.7	0.6
Group household	1.8	1.4	1.7	0.3	1.3	1.3	1.3	1.3	1.3	1.2
Other	2.8	1.3	1.6	0.3	2.5	3.0	1.0	2.6	2.4	3.1
Prefer not to say	4.0	2.2	2.4	1.4	2.9	3.3	1.1	2.4	2.8	0.9
Household Income (%)										
Below $25,000	24.2	25.5	25.8	24.2	22.6	22.7	22.5	17.0	16.7	18.0
$25,000–$40,000	18.5	19.2	16.9	27.5	19.1	17.9	23.6	15.9	15.5	17.4
$40,001 to $55,000	7.2	9.0	8.5	10.9	8.9	8.7	9.6	9.5	9.1	10.7
$55,001 to $70,000	5.2	5.8	6.1	4.8	6.1	5.6	8.1	7.1	6.8	8.3
$70,001 to $85,000	3.5	3.6	3.3	4.8	4.8	4.9	4.3	4.9	4.8	5.2
$85,001 to $100,000	2.1	3.1	3.2	2.5	3.4	3.3	3.5	4.2	4.3	3.7
$100,001 to $120,000	2.2	3.2	3.4	2.3	2.8	2.9	2.5	2.2	2.0	3.1
Over $120,000	2.8	3.4	3.5	3.0	3.6	3.4	4.5	4.6	4.6	4.6
Prefer not to say	34.4	27.2	29.1	19.9	28.7	30.6	21.4	34.8	36.2	29.1

^a^Table indicates the numbers included in each set of analyses

### Retention

Three month program retention rate was 95%; 6 month retention was 88%; 1 year retention 75%; and 3 year retention 36%. Until December 2014, 11% of withdrawals from the program were due to ill health or death.

Walker retention time according to socioeconomic characteristics in the entire registration database (*n* = 20,750 walkers, excluding 3 with no retention date data) is presented in Table 2. Participants from all SEIFA deciles were represented. Overall 14.5% of walkers reported a first language other than English, which is slightly higher than the population estimate of 10% [16]. The proportion of registrants who reported being Aboriginal or Torres Strait Islander (ATSI: 1.4%) was slightly lower than the population estimate of 3% [17]. Retention time was slightly lower among participants who were unemployed than among those who reported their employment status as other or home duties; but did not differ markedly according to gender, first language or ATSI status.

**Table 2:** Walker retention time by socioeconomic characteristics amongst current walker participants in Heart Foundation Walking (*n* = 20,750)^a^

	0-3mo (*n* = 5270) %	3-6mo (*n* = 1094) %	6-12mo (*n* = 1978) %	1-3 yr. (*n* = 6145) %	3 + yr. (*n* = 6263) %	All (*n* = 20,750) %	Years retention
							M (SD)
Gender							
Female	83.3	79.5	82.0	79.4	78.5	80.4	2.3 (2.7)
Male	16.7	20.5	18.0	20.6	21.5	19.6	2.5 (2.7)
SEIFA decile							
1: Most disadvantaged	7.3	5.4	6.3	12.0	8.8	9.1	2.6 (2.3)
2	9.7	9.8	6.6	8.5	11.2	9.5	2.7 (2.6)
3	6.6	7.8	6.9	8.6	8.2	7.9	2.5 (2.3)
4	11.3	10.6	12.2	13.6	10.3	11.8	2.4 (2.4)
5	10.4	13.7	10.2	12.2	9.8	10.9	2.3 (2.3)
6	10.2	9.9	14.9	10.4	9.3	10.4	2.5 (2.7)
7	16.3	10.3	12.1	9.3	9.0	10.9	2.1 (2.5)
8	9.6	8.6	9.4	9.3	14.5	11.0	3.3 (3.3)
9	9.3	13.6	11.1	8.2	9.0	9.3	2.4 (2.6)
10: Least disadvantaged	9.4	10.1	10.2	7.9	9.9	9.2	2.9 (3.2)
Occupation							
Employed	22.3	22.3	21.7	21.0	18.8	20.8	2.2 (2.5)
Self-employed	3.0	3.8	4.6	4.2	3.6	3.7	2.2 (2.2)
Semi-retired	4.8	5.5	5.4	5.2	4.1	4.8	2.0 (2.0)
Retired	48.9	46.9	44.2	47.0	47.7	47.4	2.3 (2.6)
Student	1.5	2.4	3.0	2.0	1.7	1.9	2.0 (2.0)
Home duties	5.9	8.6	11.0	10.0	10.4	9.1	2.8 (2.9)
Unemployed	4.6	3.2	3.8	3.5	2.5	3.5	1.7 (2.1)
Other	9.1	7.3	6.3	7.2	11.2	8.8	3.1 (3.5)
English first language							
Yes	75.5	92.0	87.4	91.9	85.8	85.5	2.3 (2.5)
No	24.5	8.0	12.6	8.1	14.2	14.5	2.5 (3.6)
Aboriginal or Torres Strait Islander							
Yes	1.4	0.7	0.8	1.1	1.9	1.4	2.6 (2.3)
No	98.6	99.3	99.2	98.9	98.1	98.6	2.4 (2.7)

^a^Table indicates the numbers included in each set of analyses

Most participants (42%) heard about the program via word of mouth, 18% via traditional media (TV, radio, newspaper, flyer), 11% from a health professional, and 9% internet/social media. Retention duration was longer among those who initially joined as the result of word of mouth (2.5y, SD 2.6) and traditional media (2.5y, SD 2.5) than among those recruited via a health professional (2.1y, SD 1.8) or internet/social media (1.3y, SD 1.5), *p* < 0.01.

### Health and physical activity levels

Table 3 presents the health characteristics and physical activity levels of participants. Approximately 60% of walkers were overweight or obese each year, which is close to the population estimate (62.8%) in the most recent Australian Health Survey [18]. Many walkers (69–81%) reported at least one chronic disease or risk factor, most commonly arthritis, high blood pressure, high cholesterol and back pain. In comparison with the general population, Australian Health Survey data for 2011–12 show lower population prevalences of comparable conditions. Almost 15% of the Australian population report arthritis, 13% back problems, 10% hypertensive disease, 10% asthma, 5% heart or vascular disease, or had suffered a stroke, and 4.6% diabetes [18, 19].

**Table 3:** Weight and health characteristics of walkers participating in each Heart Foundation Walking survey^a^

	2010 (*n* = 1984)	2011 (*n* = 2796)			2012 (*n* = 3585)			2015 (*n* = 1601)		
	All	All	Female	Male	All	Female	Male	All	Female	Male
BMI (mean, SD)	27.0 (5.7)	27.1 (5.5)	26.9 (5.5)	28.1 (5.4)	27.0 (5.5)	26.8 (5.6)	27.8 (5.2)	26.9 (5.1)	26.6 (5.0)	27.7 (5.0)
BMI category (%)^b^										
Not overweight	41.1	39.8	42.9	27.9	39.6	42.6	29.2	40.5	43.0	31.7
Overweight	35.5	36.2	34.2	43.5	38.3	36.5	44.8	36.2	34.4	42.3
Obese	23.5	24.1	22.9	28.6	22.0	20.9	26.0	23.4	22.6	26.0
Chronic disease (%)										
Arthritis	36.2	43.2	45.2	35.2	43.1	45.2	35.4	37.5	38.9	32.3
Asthma	12.3	12.5	12.8	10.8	11.9	12.1	11.4	11.1	11.6	9.0
Back pain	14.6	18.8	18.6	19.4	17.8	17.9	17.5	15.0	15.0	14.9
Respiratory disease	3.6	4.2	3.7	5.9	3.9	3.6	4.8	3.8	4.0	3.1
High blood pressure	31.3	35.3	33.9	40.3	36.0	35.1	39.6	28.1	26.6	33.9
High cholesterol	27.4	27.7	27.6	27.9	26.3	26.6	25.1	20.0	20.0	20.2
Heart disease	8.8	11.1	8.0	22.6	10.7	8.0	21.1	8.9	6.6	18.0
Diabetes	10.0	10.5	8.8	16.5	10.8	8.6	18.6	8.7	6.9	15.5
Any one (or more) of the above conditions/risk factors (%)^c^	73.5	79.5	78.4	83.1	80.9	80.2	83.5	69.3	67.8	74.8
2 or more conditions/risk factors	41.6	46.9	45.7	50.8	45.9	44.9	49.9	38.1	37.9	39.1
3 or more conditions/risk factors	18.4	23.0	21.9	26.8	21.8	21.2	24.2	17.3	16.4	20.5
Physical activity min/week (mean, SD)	Not in survey	537.6 (556.3)	521.0 (536.8)	597.4 (618.7)	541.6 (532.6)	532.6 (533.1)	569.1 (532.2)	541.7 (573.1)	528.5 (556.0)	592.2 (632.8)
Walking min/week (mean, SD)	Not in survey	241.8 (257.5)	241.3 (261.5)	246.3 (244.5)	229.7 (226.4)	229.7 (232.3)	229.4 (205.1)	258.1 (262.4)	254.9 (261.0)	270.7 (267.8)
Meeting physical activity guidelines^d^ (%)	Not in survey	70.2	69.7	72.3	70.9	70.1	73.0	71.3	70.8	73.2

^a^Table indicates the numbers included in each set of analyses

^b^Based on WHO cutpoints [31]

^c^Categories 1 or more, 2 or more and 3 or more not mutually exclusive and hence may sum to >100%

^d^Defined as the accumulation of at least 150 min of activity and at least 5 sessions of activity over 1 week

A separate analysis of the local community persons who organized the walks (data not shown) indicated that Walk Organisers had comparable sociodemographic profiles to those of participant walkers. The average BMI of Walk Organizers was also comparable to that of participant walkers at around 27 kg/m2 at every survey year. The proportions of Walk Organisers reporting at least one chronic disease or risk factor were slightly lower than those amongst walkers, though still high (57–69%), with around a third reporting two or more conditions or risk factors.

Walkers spent an average of about 9 h per week engaged in physical activity overall and 4 h per week walking. Approximately 70% of walkers met physical activity guidelines. Walk Organisers tended to have higher levels of physical activity (mean ~11 h/week), walking (~5 h/week), and adherence to guidelines (80%) (data not shown).

### Motivators for joining and maintaining participation

Table 4 shows various reasons for joining Heart Foundation Walking. Though respondents were permitted to select numerous reasons, improving fitness, health and weight loss were some of the more popular initial motivational factors for joining. Meeting new people became a more important motivator for joining over the survey years. The main reason that participants reported maintaining participation was the social aspect, particularly among women. Commonly reported ‘other’ responses included to support/accompany a spouse/friend; to walk the dog; or for mental wellbeing. Across the survey years, the walker recognition scheme, newsletters and special events were perceived as somewhat or very important by on average 69, 70 and 70% of the sample respectively.

**Table 4:** Main reasons walkers joined/still participate in Heart Foundation Walking by year^a^

	2010 (*n* = 1984)	2011 (*n* = 2796)			2012 (*n* = 3585)		2015 (*n* = 1601)			
		All	Female	Male	All	Female	Male	All	Female	Male
Main reasons JOINED (%)^b^										
To manage my weight	45.4	53.4	53.9	52.1	50.8	50.6	51.1	46.7	47.7	42.8
To meet new people	48.8	55.4	56.7	50.4	59.0	60.7	52.8	62.2	63.6	56.6
To improve my fitness	76.8	85.8	86.0	84.6	82.2	82.7	81.0	81.0	81.7	78.3
To have time out from other commitments (at 2010 this was phrased as “to have time to myself”)	8.3	19.9	21.2	14.6	20.4	21.5	15.6	17.4	18.5	13.1
To look and feel good	44.7	39.0	40.9	31.8	33.8	35.3	27.4	28.7	29.9	24.2
To improve my health	67.1	79.9	79.4	81.9	81.9	81.5	83.2	80.2	79.4	83.5
To relieve and manage stress	19.3	29.8	32.0	21.1	29.9	31.6	22.9	29.0	30.9	21.4
For fun / enjoyment	45.8	60.4	61.7	55.6	57.2	58.1	53.6	59.1	60.6	53.5
To have more energy	33.2	42.2	44.3	33.3	38.9	40.9	31.5	35.5	38.5	23.9
Because a doctor / health professional recommended me	8.3	13.7	12.0	19.8	12.3	11.0	16.4	8.8	8.1	11.6
To balance other things I do (e.g. over-indulgence)	4.8	11.9	11.5	13.2	10.8	10.8	10.5	8.1	7.9	8.9
To spend time with others	41.1	46.6	48.4	39.5	42.7	43.6	39.3	41.6	42.4	38.5
Other	6.8	2.6	2.7	2.1	3.7	3.7	3.7	4.7	4.6	5.2
Unsure	0.2	0.1	0.1	0.4	0.1	0.1	0.0	0.4	0.3	0.6
Main reasons STILL PARTICIPATING^c^ (Not asked in 2010) (%)										
Social aspect	–	61.4	64.2	51.2	56.7	58.7	49.8	73.4	75.9	64.4
Fitness / exercise	–	54.7	54.9	54.9	53.5	54.1	52.0	55.0	53.7	59.5
Health	–	25.2	25.1	23.8	26.4	24.9	31.5	21.5	21.0	23.2
Wellbeing	–	2.7	2.9	1.4	7.1	7.0	6.8	2.0	1.9	2.5
Manage weight / weight loss	–	6.7	6.5	6.9	5.1	5.3	3.9	2.5	2.2	3.2
Support / motivation	–	9.0	9.2	8.6	10.0	10.8	7.3	12.3	13.4	8.5
Walking locations and condition (e.g. safe, weather)	–	7.3	7.1	7.9	6.8	7.7	3.8	7.8	8.6	4.9
Enjoyment	–	12.0	12.9	8.6	21.6	22.8	17.0	12.3	13.1	9.5
Other	–	6.7	6.7	7.2	2.6	2.4	3.4	7.9	7.5	9.2

^a^Table indicates the numbers included in each set of analyses

^b^Percentages reflect numbers reporting each reason; these do not sum to 100% since multiple reasons could be provided

^c^Open-ended question with key responses coded into categories; again these do not sum to 100% since multiple reasons could be provided

## Discussion

Heart Foundation Walking is a large, free, community walking program that is unique in its scale and population reach, engaging more than 22,000 registered participants at the time of this evaluation. The program reached and retained a large number of participants, including vulnerable groups. The program had particularly high reach in remote and sparsely populated regions where physical activity facilities and programs are likely to be limited. Retention rates compare favourably to others reported in the scientific literature [20, 21].

The recruitment success of walking groups is often measured by the numbers of participants joining, rather than the reach to those who stand to benefit most [22]. However, walking groups have the potential to widen health inequities if they are not sensitively targeted to reach and cater to the needs of these high-risk groups [7] including women, people who are socioeconomically disadvantaged, older adults, adults who are overweight or obese, and people with, or at risk of, chronic disease, for whom physical activity may be particularly beneficial, but who may face additional health-related barriers to being active [23]. The data show that the Heart Foundation Walking program had good impact in attracting women; older adults; those who are socioeconomically disadvantaged; and those who have one or more chronic diseases or risk factors. The program also attracted a higher than average proportion of participants who live alone. As not all walkers completed surveys, these data are illustrative rather than comprehensive. Given typically lower than average response rates in mail surveys among those who are socioeconomically disadvantaged [24, 25], for example, these data may under-estimate the true reach of the walking program to these individuals.

The repeat cross-sectional design and lack of a control group preclude strong conclusions regarding particular elements that contributed to the favourable reach and retention rates. Nonetheless, the data describe the elements of one successful model, and suggest some features that may be implemented in future programs. These include the sponsorship by a well-known national organisation; targeting regions that may lack services or comparable initiatives; the use of inexpensive wide-reaching recruitment modes; and facilitating social interactions, which appear to be of increasing importance to participants. Given the perceived importance of low-cost walker recognition schemes, communications and special events by the majority of participants, incorporating these elements into future programs would be warranted. Investigating the reasons for continued participation amongst those who did not rate these features as important could also add to our understanding of how future programs might enhance retention. Future programs could also adopt the volunteer model used here, which builds community capacity and reduces costs by engaging and supporting local volunteers as Walk Organisers. Organisers were more physically active but otherwise had comparable characteristics (sociodemographics, BMI) to participants, hence potentially serving as relatable role models. Analyses of retention times suggest that future programs might focus recruitment efforts on traditional media and encouraging existing participants to recruit others.

Typically, participant drop-out rates present a major concern in community health programs. Substantial heterogeneity makes it difficult to directly compare reach and retention rates across different programs. Despite data gaps, existing reports of broadly comparable programs show that 30–76% of people who begin a new exercise program will drop out within 1 year [20, 21, 26]. The greatest attrition typically occurs in the first 3 months (e.g., 36%) [21], and approximately 50% within 6 months [27]. Results from the present study, showing an average 6-month retention of 88%, 1-year retention of 75%, and average participation duration of 2.4 years among Walkers, compare favourably to the rates reported in the limited available literature. Importantly, voluntary attrition was even lower than the rates reported here, which include non-voluntary drop-out (due to illness or death).

A study of a similar community-based program reported by Jancey and colleagues [20] reported a 6 month retention of 65% (i.e. 35% attrition) among adults aged 65–74 years. Unlike Heart Foundation Walking, retention was poorer among those from disadvantaged areas, and those who were obese, or insufficiently active. Program differences that may explain the comparatively higher retention rates of Heart Foundation Walking include the fact that, unlike Jancey et al. [20], Heart Foundation Walking was designed as a long-term program, fostered word-of-mouth recruitment, and fostered community engagement via recruitment and ongoing investment in volunteers.

Around 70% of Walkers and 80% of Organisers were meeting physical activity recommendations. The most recent population prevalence data suggest that only 43% of Australian adults are meeting recommendations [2]. Notably, it was not possible to determine whether Heart Foundation Walking contributed to these high levels of physical activity or whether the program attracted participants who were more active. However, a previous evaluation of a state-based program suggested that participation for 12 months increased both walking and total physical activity, particularly among initially inactive participants [28].

The data presented suggest that the reasons participants joined Heart Foundation Walking were different from the reasons participants continued with the program. Improving fitness and health appeared to be the most important motivators for joining the program whereas the social aspect was the strongest motivator for continuing. Social engagement may be particularly relevant for walking, as opposed to more vigorous or structured group-based activity such as exercise classes, which typically do not offer the same opportunities to talk with other participants during the activity. These data corroborate findings from a meta-analysis showing the most consistent predictor of participation in physical activity programs among socioeconomically disadvantaged women was a social component [29]. This highlights the importance of focusing on building, strengthening and maintaining social networks that support behaviour change [3], particularly given the documented challenges in maintaining behaviour change [5]. In order to attract participants, future walking programs could implement tailored recruitment campaigns that promote benefits to fitness and health as well as opportunities to meet and spend time with others. In light of the finding that few participants were motivated to join as a result of a health professional recommendation, a ‘bring a friend’ initiative might be a more useful promotional approach. Considering the importance of social factors in retaining participants, programs might also consider activities that enhance opportunities for social engagement, such as walks linked with lunches or picnic days. With the increasing proliferation of mobile phone technology, provision of ‘virtual’ support could also be trialled, for example through online challenges and Facebook/social media support groups, to further facilitate social connectedness among participants. Mobile technology could also be used to provide motivational strategies, such as text message congratulations on significant milestones, or ‘we miss you’ messages for those who haven’t walked for some time. The impact and cost-effectiveness of such strategies could be evaluated in future studies.

Capitalising on the unique evaluation of a large, nationwide physical activity program in a real-world setting, this study utilised data from sizeable numbers of respondents over four survey points to explore reach, retention, participant characteristics and motivators for participation in a national walking group program. Notwithstanding these strengths, the study was limited by its reliance on self-report measures collected with tools that have not been tested for validity or reliability among this study population; and use of a repeat cross-sectional design which meant that temporal sequencing could not be determined. We also did not have access to data on frequency of attendance, and did not measure actual health benefits. It is possible that survey respondents were not representative of Heart Foundation Walking participants more generally. In particular the response rate at 2015 was low, potentially reflecting the shift to email-only administration, given typically lower responses to online than paper-based surveys [30].

## Conclusions

**Table:** 

Elements of success
Auspiced by a well-established reputable national non-government organisation
Free for members
Large reach with groups established nationwide
Embedded in and supported by local community
Volunteer-led by community members
Capacity building
Social opportunities
Socially-based promotion (members recruiting members)
Retention strategies including member incentives

Walking is a popular activity with health benefits and a low risk of injury that requires little skill or equipment. This study suggests that free group-based walking programs are a promising approach for increasing population levels of physical activity and attracting participants at high risk of inactivity. This study provides a model for such a program, with key elements (summarised in the Box) including backing of a well-known national non-government organisation; support of local government and community health staff; community capacity building via local volunteer Walk Organisers; low-cost and word of mouth promotion; retention strategies including walker recognition schemes, newsletters and annual/special events; and opportunities for social interaction. Though initial motivation factors may include health and fitness, programs should market social aspects as they appear to be an especially important component of group-based walking, particularly for continued participation.

## Abbreviations

ATSI: Aboriginal or Torres Strait Islander
BMI: Body mass index (kg/m^2^)
SA4: Statistical Areas Level 4
SEIFA: Socioeconomic Index for Areas
